# True 4D Image Denoising on the GPU

**DOI:** 10.1155/2011/952819

**Published:** 2011-10-01

**Authors:** Anders Eklund, Mats Andersson, Hans Knutsson

**Affiliations:** ^1^Division of Medical Informatics, Department of Biomedical Engineering, Linköping University, Linköping, Sweden; ^2^Center for Medical Image Science and Visualization (CMIV), Linköping University, Linköping, Sweden

## Abstract

The use of image denoising techniques is an important part of many medical imaging applications. One common application is to improve the image quality of low-dose (noisy) computed tomography (CT) data. While 3D image denoising previously has been applied to several volumes independently, there has not been much work done on true 4D image denoising, where the algorithm considers several volumes at the same time. The problem with 4D image denoising, compared to 2D and 3D denoising, is that the computational complexity increases exponentially. In this paper we describe a novel algorithm for true 4D image denoising, based on local adaptive filtering, and how to implement it on the graphics processing unit (GPU). The algorithm was applied to a 4D CT heart dataset of the resolution 512  × 512  × 445  × 20. The result is that the GPU can complete the denoising in about 25 minutes if spatial filtering is used and in about 8 minutes if FFT-based filtering is used. The CPU implementation requires several days of processing time for spatial filtering and about 50 minutes for FFT-based filtering. The short processing time increases the clinical value of true 4D image denoising significantly.

## 1. Introduction

Image denoising is commonly used in medical imaging in order to help medical doctors to see abnormalities in the images. Image denoising was first applied to 2D images [1–3] and then extended to 3D data [4–6], 3D data can either be collected as several 2D images over time or as one 3D volume. A number of medical imaging modalities (e.g., computed tomography (CT), ultrasound (US) and magnetic resonance imaging (MRI)) now provide the possibility to collect 4D data, that is, time-resolved volume data. This makes it possible to, for example, examine what parts of the brain that are active during a certain task (functional magnetic resonance imaging (fMRI)). While 4D CT data makes it possible to see the heart beat in 3D, the drawback is that a lower amount of X-ray exposure has to be used for 4D CT data collection, compared to 3D CT data collection, in order to not harm the patient. When the amount of exposure is decreased, the amount of noise in the data increases significantly.

Three-dimensional image denoising has previously been applied to several time points independently, but there has not been much work done on *true* 4D image denoising where the algorithm considers several volumes at the same time (and not a single volume at a time). Montagnat et al. [7] applied 4D anisotropic diffusion filtering to ultrasound volumes and Jahanian et al. [8] applied 4D wavelet denoising to diffusion tensor MRI data. For CT data, it can be extra beneficial to use the time dimension in the denoising, as some of the reconstruction artefacts vary with time. It is thereby possible to remove these artefacts by taking full advantage of the 4D data. While true 4D image denoising is very powerful, the drawback is that the processing time increases exponentially with respect to dimensionality.

The rapid development of graphics processing units (GPUs) has resulted in that many algorithms in the medical imaging domain have been implemented on the GPU, in order to save time and to be able to apply more advanced analysis. To give an example of the rapid GPU development, a comparison of three consumer graphic cards from Nvidia is given in Table 1. The time frame between each GPU generation is 2-3 years. Some examples of fields in medical imaging that have taken advantage of the computational power of the GPU are image registration [9–13], image segmentation [14–16] and fMRI analysis [17–20].

In the area of image denoising, some algorithms have also been implemented on the GPU. Already in 2001 Rumpf and Strzodka [21] described how to apply anisotropic diffusion [3] on the GPU. Howison [22] made a comparison between different GPU implementations of anisotropic diffusion and bilateral filtering for 3D data. Su and Xu [23] in 2010 proposed how to accelerate wavelet-based image denoising by using the GPU. Zhang et al. [24] describe GPU-based image manipulation and enhancement techniques for dynamic volumetric medical image visualization, but enhancement in this case refers to enhancement of the visualization, and not of the 4D data. Recently, the GPU has been used for real-time image denoising. In 2007, Chen et al. [25] used bilateral filtering [26] on the GPU for real-time edge-aware image processing. Fontes et al. [27] in 2011 used the GPU for real-time denoising of ultrasound data and Goossens et al. [28] in 2010 managed to run the commonly used nonlocal means algorithm [29] in real time.

To our knowledge, there has not been any work done about true 4D image denoising on the GPU. In this work we therefore present a novel algorithm, based on local adaptive filtering, for 4D denoising and describe how to implement it on the GPU, in order to decrease the processing time and thereby significantly increase the clinical value.

## 2. Methods

 In this section, the algorithm that is used for true 4D image denoising will be described.

### 2.1. The Power of Dimensionality

**Figure 1:**
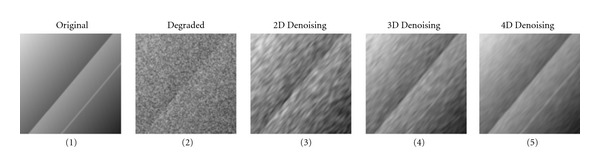
(1) Original test image without noise. There is a large step in the middle, a bright thin line and a shading from the top left corner to the bottom right corner. (2) Original test image with a lot of noise. The step is barely visible, while it is impossible to see the line or the shading. (3) Resulting image after 2D denoising. The step is almost visible and it is possible to see that the top left corner is brighter than the bottom right corner. (4) Resulting image after 3D denoising. Now the step and the shading are clearly visible, but not the line. (5) Resulting image after 4D denoising. Now all parts of the image are clearly visible.

To show how a higher number of dimensions, the power of dimensionality, can improve the denoising result, a small test is first conducted on synthetic data. The size of the 4D data is 127 × 127 × 9 × 9, but there is no signal variation in the last two dimensions. The data contains a large step, a thin line, and a shading from the top left corner to the bottom right corner. A large amount of 4D additive noise was finally added to the data. Image denoising of different dimensionality was then applied. For the 2D case, the denoising was done on one 127 × 127 image, for the 3D case, the denoising was done on one 127 × 127 × 9 volume and for the 4D case all the data was used. A single anisotropic lowpass filter was used for the denoising, and the filter had the same dimensionality as the data and was oriented along the structures. The original test data, the test data with noise and the denoising results are given in Figure 1. It is clear that the denoising result is improved significantly for each new dimension.

### 2.2. Adaptive Filtering in 4D

The denoising approach that our work is based on is adaptive filtering. It was introduced for 2D by Knutsson et al. in 1983 [2] and then extended to 3D in 1992 [4]. In this work, the same basic principles are used for adaptive filtering in 4D. The main idea is to first estimate the local structure tensor [30] (by using a first set of filters) in each neighbourhood of the data and then let the tensor control the reconstruction filters (a second set of filters). The term reconstruction should in this paper not be confused with the reconstruction of the CT data. The local structure tensor **T** is in 4D a 4 × 4 symmetric matrix in each time voxel,

(1) $$\begin{array}{c} T=\left(\begin{array}{cccc} t_{1} & t_{2} & t_{3} & t_{4}\\ t_{2} & t_{5} & t_{6} & t_{7}\\ t_{3} & t_{6} & t_{8} & t_{9}\\ t_{4} & t_{7} & t_{9} & t_{10} \end{array}\right)=\left(\begin{array}{cccc} xx & xy & xz & xt\\ xy & yy & yz & yt\\ xz & yz & zz & zt\\ xt & yt & zt & tt \end{array}\right), \end{array}$$

and contains information about the local structure in the data, that can be used to control the weights of the reconstruction filters. The result of the adaptive filtering is that smoothing is done along structures (such as lines and edges in 2D), but not perpendicular to them.

### 2.3. Adaptive Filtering Compared to Other Methods for Image Denoising

Compared to more recently developed methods for image denoising (e.g., nonlocal means [29], anisotropic diffusion [3] and bilateral filtering [26]), adaptive filtering is in our case used for 4D image denoising for three reasons. First, adaptive filtering is computationally more efficient than the other methods. Nonlocal means can give very good results, but the algorithm can be extremely time consuming (even if GPUs are used). Anisotropic diffusion is an *iterative* algorithm and can therefore be rather slow. Adaptive filtering is a *direct* method that does not need to be iterated. Bilateral filtering does not only require a multiplication for each filter coefficient and each data value, but also an evaluation of the intensity range function (e.g., an exponential) which is much more expensive to perform than a multiplication. Second, the tuning of the parameters is for our denoising algorithm rather easy to understand and to explore. When a first denoising result has been obtained, it is often obvious how to change the parameters to improve the result. This is not always the case for other methods. Third, the adaptive filtering approach has been proven to be very robust (it is extremely seldom that a strange result is obtained). Adaptive filtering has been used for 2D image denoising in commercial clinical software for over 20 years and a recent 3D study [31] proves its potential, robustness, and clinical acceptance. The nonlocal means algorithm only works if the data contains several neighbourhoods with similar properties.

### 2.4. Estimating the Local Structure Tensor Using Quadrature Filters

The local structure tensor can, for example, be estimated by using quadrature filters [5, 30]. Quadrature filters *Q* are zero in one half of the frequency domain (defined by the direction of the filter) and can be expressed as two polar separable functions, one radial function *R* and one directional function *D*,

(2) $$\begin{array}{c} Q\left(u\right)=R\left(||u||\right)D\left(u\right), \end{array}$$

where **u** is the frequency variable. The radial function is a lognormal function

(3) $$\begin{array}{c} R\left(||u||\right)=\exp\;\left(C\;{\ln\;}^{2}\left(\frac{||u||}{u_{0}}\right)\right),\;C=\frac{-4}{B^{2}\ln\;\left(2\right)}, \end{array}$$

where *u*_0_ is the centre frequency of the filter and *B* is the bandwidth (in octaves). The directional function depends on the angle *θ* between the filter direction vector $\hat{n}$ and the normalized frequency coordinate vector **u** as cos (*θ*)^2^,

(4) $$\begin{array}{c} D\left(u\right)=\{\begin{array}{cc} {\left(u^{T}\hat{n}\right)}^{2}, & u^{T}\hat{n}>0,\\ 0, & \text{otherwise}. \end{array} \end{array}$$

Quadrature filters are Cartesian nonseparable and complex valued in the spatial domain, the real part is even and in 2D acts as a line detector, while the imaginary part is odd and in 2D acts as an edge detector. In 3D, the even and odd filters correspond to a plane detector and a 3D edge detector. In 4D, the plane and 3D edge may in addition be time varying. The complex-valued filter response *q* is an estimate of a bandpass filtered version of the analytical signal with magnitude *A* and phase *ϕ*,

(5) $$\begin{array}{c} q=A\left(\cos\;\left(\phi \right)+i\cdot \sin\;\left(\phi \right)\right)=Ae^{i\phi }. \end{array}$$

The tensor is calculated by multiplying the magnitude of the quadrature filter response *q*_*k*_ with the outer product of the filter direction vector ${\hat{n}}_{k}$ and then summing the result over all filters *k*,

(6) $$\begin{array}{c} T=\overset{N_{f}}{\underset{k=1}{\sum }}\left|q_{k}\right|\left(c_{1}{\hat{n}}_{k}{\hat{n}}_{k}^{T}-c_{2}I\right), \end{array}$$

where *c*_1_ and *c*_2_ are scalar constants that depend on the dimensionality of the data [5, 30], *N*_*f*_ is the number of quadrature filters and **I** is the identity matrix. The resulting tensor is *phase invariant*, as the magnitude of the quadrature filter response is invariant to the type of local neighbourhood (e.g., in 2D bright lines, dark lines, dark to bright edges, etc.). This is in contrast to when the local structure tensor is estimated by using gradient operators, such as Sobel filters.

The number of filters that are required to estimate the tensor depends on the dimensionality of the data and is given by the number of independent components of the symmetric local structure tensor. The required number of filters is thus 3 for 2D, 6 for 3D and 10 for 4D. The given tensor formula, however, assumes that the filters are evenly spread. It is possible to spread 6 filters evenly in 3D, but it is not possible to spread 10 filters evenly in 4D. For this reason, 12 quadrature filters have to be used in 4D (i.e., a total of 24 filters in the spatial domain, 12 real valued and 12 complex valued). To apply 24 nonseparable filters to a 4D dataset requires a huge number of multiplications. In this paper a new type of filters, *monomial* filters [32], are therefore used instead.

### 2.5. Estimating the Local Structure Tensor Using Monomial Filters

Monomial filters also have one radial function *R* and one directional function *D*. The directional part of the monomial filters are products of positive integer powers of the components of the frequency variable **u**. The monomial filter matrices of order one, **F**_1_, and two, **F**_2_, are in the frequency domain defined as

(7) $$\begin{array}{c} F_{1,n}=R\left(||u||\right){\hat{u}}_{n},\;\;F_{2,mn}=R\left(||u||\right){\hat{u}}_{m}{\hat{u}}_{n}. \end{array}$$

The monomial filters are first described for 2D and then generalized to 4D.

#### 2.5.1. Monomial Filters in 2D

**Figure 2:**
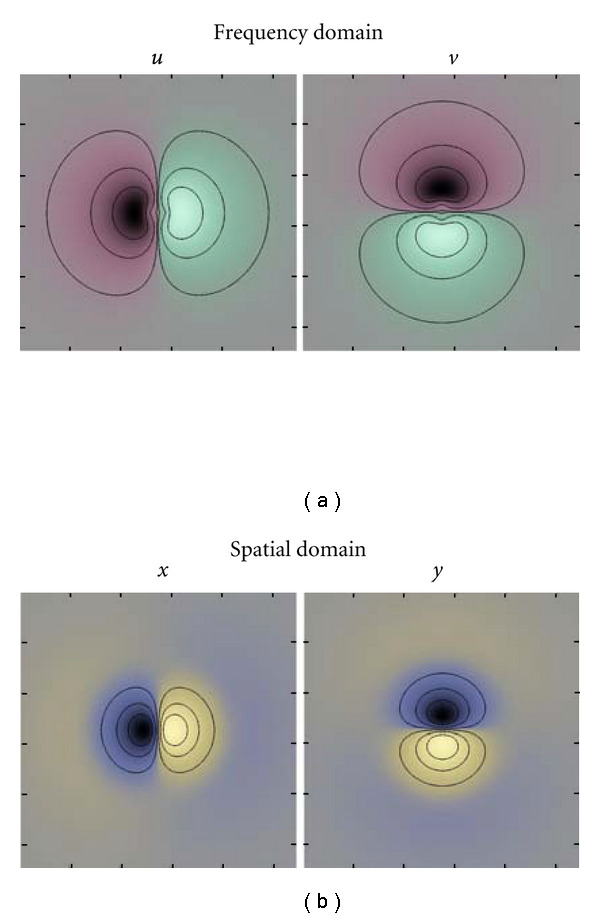
(a) Two-dimensional monomial filters (*u*, *v*), of the first order, in the frequency domain. Green indicates positive real values and red indicates negative real values. The black lines are isocurves. (b) Two-dimensional monomial filters (*x*, *y*), of the first order, in the spatial domain. Yellow indicates positive imaginary values, and blue indicates negative imaginary values. Note that these filters are odd and imaginary.

**Figure 3:**
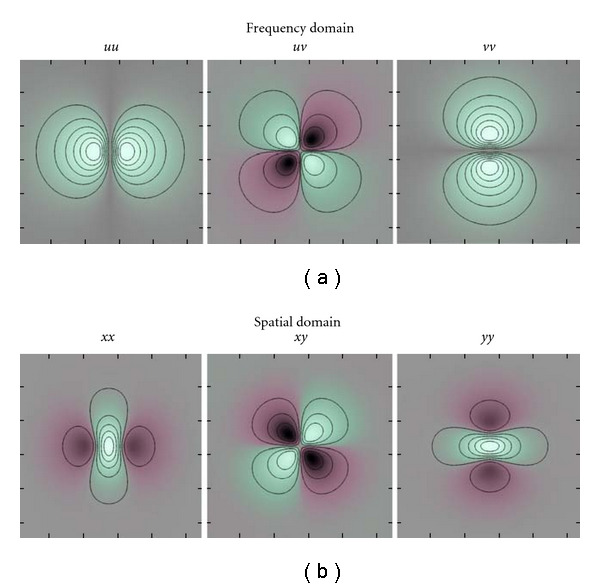
(a) Two-dimensional monomial filters (*uu*, *uv*, *v*), of the second order, in the frequency domain. Green indicates positive real values, and red indicates negative real values. The black lines are isocurves. (b) Two-dimensional monomial filters (*xx*, *xy*, *yy*), of the second order, in the spatial domain. Green indicates positive real values, and red indicates negative real values. Note that these filters are even and real.

In 2D, the frequency variable is in this work defined as **u** = [*u*  *v*]^*T*^. The directional part of first-order monomial filters are *x*, *y* in the spatial domain and *u*, *v* in the frequency domain. Two-dimensional monomial filters of the first-order are given in Figure 2. The directional part of second-order monomial filters are *xx*, *xy*, *yy* in the spatial domain and *uu*, *uv*, *vv* in the frequency domain. Two dimensional monomial filters of the second order are given in Figure 3. 

The monomial filter response matrices **Q** are either calculated by convolution in the spatial domain or by multiplication in the frequency domain. For a simple signal with phase *θ* (e.g., **s**(**x**) = *A*cos (**u**^*T*^**x** + *θ*)); the monomial filter response matrices of order one and two can be written as

(8) $$\begin{array}{c} Q_{1}=-iA\sin\;\left(\theta \right){\left[uv\right]}^{T},\\ Q_{2}=A\cos\;\left(\theta \right)\left(\begin{array}{cc} uu & uv\\ uv & vv \end{array}\right). \end{array}$$

The first-order products are odd functions and are thereby related to the odd sine function, the second order products are even functions and are thereby related to the even cosine function (note the resemblance with quadrature filters that have one even real part and one odd imaginary part). By using the fact that *u*^2^ + *v*^2^ = 1, the outer products of the filter response matrices give

(9) $$\begin{array}{c} Q_{1}{Q_{1}}^{T}={\sin\;}^{2}\left(\theta \right){\left|A\right|}^{2}\left(\begin{array}{cc} uu & uv\\ uv & vv \end{array}\right),\\ Q_{2}{Q_{2}}^{T}={\cos\;}^{2}\left(\theta \right){\left|A\right|}^{2}\left(\begin{array}{cc} uu & uv\\ uv & vv \end{array}\right). \end{array}$$

The local structure tensor **T** is then calculated as

(10) $$\begin{array}{c} T=Q_{1}{Q_{1}}^{T}+Q_{2}{Q_{2}}^{T}={\left|A\right|}^{2}\left(\begin{array}{cc} uu & uv\\ uv & vv \end{array}\right). \end{array}$$

From this expression, it is clear that the estimated tensor, as previously, is phase invariant as the square of one odd part and the square of one even part are combined. For information about how to calculate the tensor for higher-order monomials, see our recent work [32].

#### 2.5.2. Monomial Filters in 4D

A total of 14 nonseparable 4D monomial filters (4 odd of the first-order (*x*, *y*, *z*, *t*) and 10 even of the second-order (*xx*, *xy*, *xz*, *xt*, *yy*, *yz*, *yt*, *zz*, *zt*, *tt*)) with a spatial support of 7 × 7 × 7 × 7 time voxels are applied to the CT volumes. The filters have a lognormal radial function with centre frequency 3*π*/5 and a bandwidth of 2.5 octaves. The filter kernels were optimized with respect to ideal frequency response, spatial locality, and expected signal-to-noise ratio [5, 33].

By using equation (10) for the 4D case, and replacing the frequency variables with the monomial filter responses, the 10 components of the structure tensor are calculated according to

(11) $$\begin{array}{cc} t_{1} & =fr_{1}\cdot fr_{1}+fr_{5}\cdot fr_{5}+fr_{6}\cdot fr_{6}+fr_{7}\cdot fr_{7}\\ & \;+fr_{8}\cdot fr_{8},\\ t_{2} & =fr_{1}\cdot fr_{2}+fr_{5}\cdot fr_{6}+fr_{6}\cdot fr_{9}+fr_{7}\cdot fr_{10}\\ & \;+fr_{8}\cdot fr_{11},\\ t_{3} & =fr_{1}\cdot fr_{3}+fr_{5}\cdot fr_{7}+fr_{6}\cdot fr_{10}+fr_{7}\cdot fr_{12}\\ & \;+fr_{8}\cdot fr_{13},\\ t_{4} & =fr_{1}\cdot fr_{4}+fr_{5}\cdot fr_{8}+fr_{6}\cdot fr_{11}+fr_{7}\cdot fr_{13}\\ & \;+fr_{8}\cdot fr_{14},\\ t_{5} & =fr_{2}\cdot fr_{2}+fr_{6}\cdot fr_{6}+fr_{9}\cdot fr_{9}+fr_{10}\cdot fr_{10}\\ & \;+fr_{11}\cdot fr_{11},\\ t_{6} & =fr_{2}\cdot fr_{3}+fr_{6}\cdot fr_{7}+fr_{9}\cdot fr_{10}+fr_{10}\cdot fr_{12}\\ & \;+fr_{11}\cdot fr_{13},\\ t_{7} & =fr_{2}\cdot fr_{4}+fr_{6}\cdot fr_{8}+fr_{9}\cdot fr_{11}+fr_{10}\cdot fr_{13}\\ & \;+fr_{11}\cdot fr_{14},\\ t_{8} & =fr_{3}\cdot fr_{3}+fr_{7}\cdot fr_{7}+fr_{10}\cdot fr_{10}+fr_{12}\cdot fr_{12}\\ & \;+fr_{13}\cdot fr_{13},\\ t_{9} & =fr_{3}\cdot fr_{4}+fr_{7}\cdot fr_{8}+fr_{10}\cdot fr_{11}+fr_{12}\cdot fr_{13}\\ & \;+fr_{13}\cdot fr_{14},\\ t_{10} & =f{\text{r}}_{4}\cdot fr_{4}+fr_{8}\cdot fr_{8}+fr_{11}\cdot fr_{11}+fr_{13}\cdot fr_{13}\\ & \;+fr_{14}\cdot fr_{14}, \end{array}$$

where *fr*_*k*_ denotes the filter response for monomial filter *k*. The first term relates to **Q**_1_**Q**_1_^*T*^, and the rest of the terms relate to **Q**_2_**Q**_2_^*T*^, in total **Q**_1_**Q**_1_^*T*^ + **Q**_2_**Q**_2_^*T*^.

If monomial filters are used instead of quadrature filters, the required number of 4D filters is thus decreased from 24 to 14. Another advantage is that the monomial filters require a smaller spatial support, which makes it easier to preserve details and contrast in the processing. A smaller spatial support also results in a lower number of filter coefficients, which decreases the processing time.

### 2.6. The Control Tensor

When the local structure tensor **T** has been estimated, it is then mapped to a control tensor **C**, by mapping the magnitude (energy) and the isotropy of the tensor. The purpose of this mapping is to further improve the denoising. For 2D and 3D image denoising, this mapping can be done by first calculating the eigenvalues and eigenvectors of the structure tensor in each element of the data. The mapping is first described for 2D and then for 4D.

#### 2.6.1. Mapping the Magnitude of the Tensor in 2D

**Figure 4:**
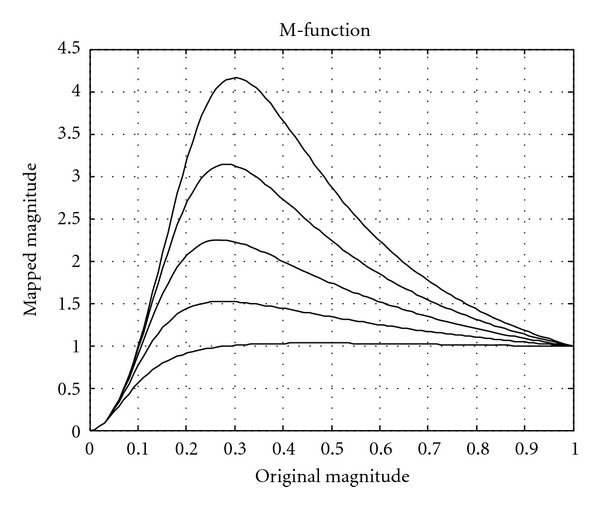
Examples of the M-function that maps the magnitude of the structure tensor. If the magnitude of the structure tensor is too low, the magnitude is set to zero for the control tensor, such that no highpass information is used in this part of the data. The overshoot is intended to amplify structures that have a magnitude that is slightly above the noise threshold.

In the 2D case, the magnitude *γ*_0_ of the tensor is calculated as

(12) $$\begin{array}{c} \gamma_{0}=\sqrt{\lambda_{1}^{2}+\lambda_{2}^{2}}, \end{array}$$

where *λ*_1_ and *λ*_2_ are the two eigenvalues. The magnitude *γ*_0_ is normalized to vary between 0 and 1 and is then mapped to *γ* with a so-called M-function according to

(13) $$\begin{array}{c} \gamma =\left(\frac{\gamma_{0}^{\;\beta }}{\gamma_{0}^{\;\alpha \;+\;\beta }+\sigma^{\;\beta }}\right), \end{array}$$

where *α*, *β*, and *σ* are parameters that are used to control the mapping. The *σ* variable is directly proportional to the signal-to-noise (SNR) ratio of the data and acts as a soft noise threshold, *α* mainly controls the overshoot (that can be used for dynamic range compression or to amplify areas that have a magnitude slightly above the noise threshold), and *β* mainly controls the slope/softness of the curve. The purpose of this mapping is to control the general usage of highpass information. The highpass information should only be used where there is a well-defined structure in the data. If the magnitude of the structure tensor is low, one can assume that the neighbourhood only contains noise. Some examples of the M-function are given in Figure 4.

#### 2.6.2. Mapping the Isotropy of the Tensor in 2D

**Figure 5:**
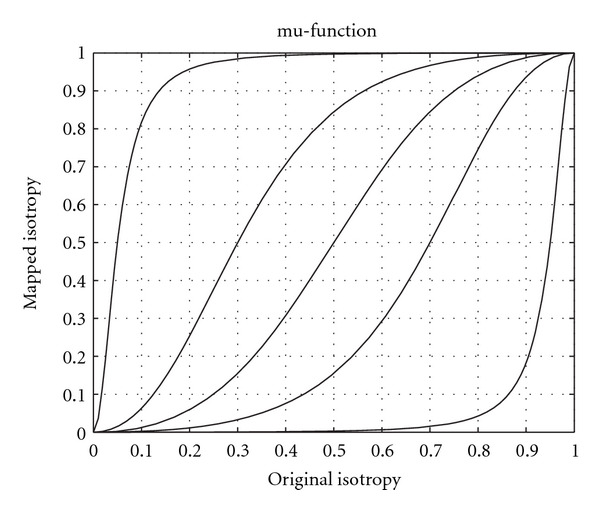
Examples of the mu-function that maps the isotropy of the structure tensor. If the structure tensor is almost isotropic (a high value on the *x*-axis) the control tensor becomes more isotropic. If the structure tensor is anisotropic (a low value on the *x*-axis) the control tensor becomes even more anisotropic.

**Figure 6:**
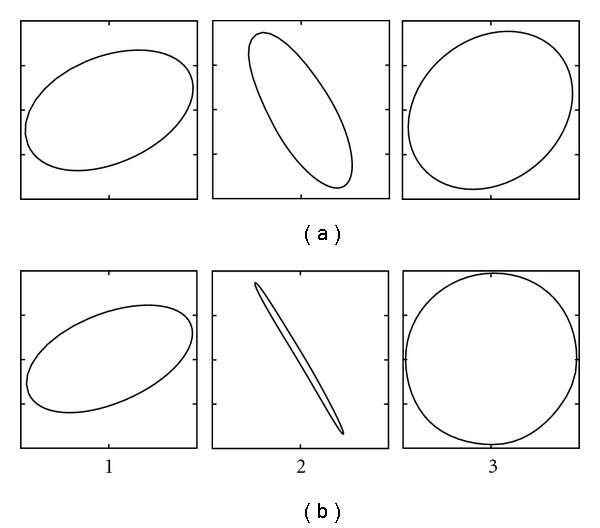
Three examples of isotropy mappings. (a) Original structure tensors. (b) Mapped control tensors. If the structure tensor is anisotropic, the control tensor becomes even more anisotropic (examples 1 and 2). If the structure tensor is almost isotropic, it becomes more isotropic (example 3).

The isotropy *ϕ*_0_ is in 2D calculated as

(14) $$\begin{array}{c} \phi_{0}=\frac{\lambda_{2}}{\lambda_{1}} \end{array}$$

and is mapped to *ϕ* with a so called mu-function according to

(15) $$\begin{array}{c} \phi =\frac{{\left(\phi_{0}\left(1-\alpha \right)\right)}^{\beta }}{{\left(\phi_{0}\left(1-\alpha \right)\right)}^{\beta }+{\left(\alpha \left(1-\phi_{0}\right)\right)}^{\beta }}, \end{array}$$

where *α* and *β* are parameters that are used to control the mapping, *α* mainly controls the transition of the curve and *β* mainly controls the slope/softness. The purpose of this mapping is to control the usage of highpass information in the nondominant direction, that is, the direction that is given by the eigenvector corresponding to the smallest eigenvalue. This is done by making the tensor more isotropic if it is slightly isotropic, or making it even more anisotropic if it is anisotropic. Some examples of the mu-function are given in Figure 5. Some examples of isotropy mappings are given in Figure 6. The M-function and the mu-function are further explained in [5].

#### 2.6.3. The Tensor Mapping in 2D

The control tensor **C** is finally calculated as

(16) $$\begin{array}{c} C=\gamma e_{1}{e_{1}}^{T}+\gamma \phi e_{2}{e_{2}}^{T}, \end{array}$$

where **e**_1_ is the eigenvector corresponding to the largest eigenvalue *λ*_1_ and **e**_2_ is the eigenvector corresponding to the smallest eigenvalue *λ*_2_. The mapping thus preserves the eigensystem, but changes the eigenvalues and thereby the shape of the tensor.

#### 2.6.4. The Complete Tensor Mapping in 4D

For matrices of size 2 × 2 and 3 × 3, there are direct formulas for how to calculate the eigenvalues and eigenvectors, but for 4 × 4 matrices, there are no such formulas and this complicates the mapping. It would of course be possible to calculate the eigenvalues and eigenvectors by other approaches, such as the power iteration algorithm, but this would be extremely time consuming as the mapping to the control tensor has to be done in each time voxel. The mapping of the local structure tensor to the control tensor is in this work therefore performed in a way that does not explicitly need the calculation of eigenvalues and eigenvectors. The tensor magnitude is first calculated as

(17) $$\begin{array}{c} T_{\text{mag}}={||T^{8}||}^{1/8}, \end{array}$$

where ||·|| denotes the Frobenius norm. The exponent will determine how close to *λ*_1_ the estimated tensor magnitude will be; a higher exponent will give better precision, but an exponent of 8 has proven to be sufficient in practice. To reduce the computational load, **T**^8^ is calculated as

(18) $$\begin{array}{cc} T^{2} & =T*T,\\ T^{4} & =T^{2}*T^{2},\\ T^{8} & =T^{4}*T^{4}, \end{array}$$

where ∗ denotes matrix multiplication. *γ*_0_ is then calculated as

(19) $$\begin{array}{c} \gamma_{0}=\sqrt{\frac{T_{\text{mag}}}{\max\;\left(T_{\text{mag}}\right)}}, \end{array}$$

where the max operator is for the entire data set, such that the maximum value of *γ*_0_ will be 1, *γ*_0_ is then mapped to *γ* by using the M-function.

To map the isotropy, the structure tensor is first normalized as

(20) $$\begin{array}{c} \hat{T}=\frac{T}{T_{\text{mag}}}, \end{array}$$

such that the tensor only carries information about the anisotropy (shape). The fact that $\hat{T}$ and $I-\hat{T}$ have the same eigensystem is used, such that the control tensor can be calculated as

(21) $$\begin{array}{c} C=\gamma \left(\phi I+\left(1-\phi \right)\cdot \hat{T}\right), \end{array}$$

where **I** is the identity matrix. The following formulas are an ad hoc modification of this basic idea, that do not explicitly need the calculation of the isotropy *ϕ* and that give good results for our CT data. The basic idea is that the ratio of the eigenvalues of the tensor change when the tensor is multiplied with itself a number of times, and thereby the shape of the tensor also changes. This approach does not give exactly the same results as the original isotropy mapping, but it circumvents the explicit calculation of eigenvalues and eigenvectors. A help variable ${\hat{T}}_{f}$ is first calculated as

(22) $$\begin{array}{c} {\hat{T}}_{f}={\hat{T}}^{2}*\left(I+2\cdot \left(I-\hat{T}\right)\right), \end{array}$$

and then the control tensor **C** is calculated as

(23) $$\begin{array}{c} C=\gamma \;\left(I-{\left(I-{\hat{T}}_{f}\right)}^{8}*\left(I+8\cdot {\hat{T}}_{f}\right)\right). \end{array}$$

**Figure 7:**
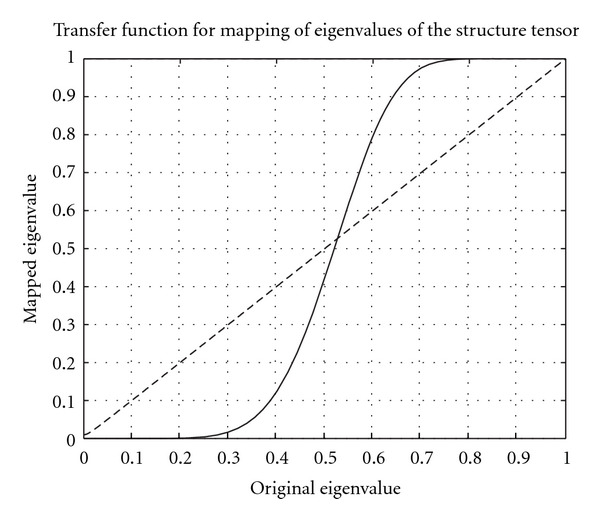
The transfer function that maps the eigenvalues of the structure tensor.

The resulting transfer function that maps each eigenvalue is given in Figure 7. Eigenvalues that are small become even smaller, and eigenvalues that are large become even larger. The result of this eigenvalue mapping is similar to the isotropy mapping examples given in Figure 6.

### 2.7. Calculating the Denoised Data

Eleven nonseparable reconstruction filters, one lowpass filter **H**_0_ of the zeroth order and 10 highpass filters **H**_2,**mn**_ of the second order, with a spatial support of 11 × 11 × 11 × 11 time voxels are applied to the CT volumes. The denoised 4D data *i*_d_ is calculated as the sum of the lowpass-filtered data, *i*_lp_, and the highpass filtered data for each highpass-filter *k*, *i*_hp(*k*)_, weighted with the components **C**_*k*_ of the control tensor **C**,

(24) $$\begin{array}{c} i_{\text{d}}=i_{\text{lp}}+\overset{10}{\underset{k=1}{\sum }}C_{k}\cdot i_{\text{hp}(k)}. \end{array}$$

The result is that the 4D data is lowpass filtered in all directions and then highpass information is put back where there is a well-defined structure. Highpass information is put back in the dominant direction of the local neighbourhood (given by the eigenvector related to the largest eigenvalue) if the tensor magnitude is high. Highpass information is put back in the nondominant directions (given by the eigenvectors related to the smaller eigenvalues) if the tensor magnitude is high and the anisotropy is low.

### 2.8. The Complete Algorithm

All the processing steps of the denoising algorithm are given in Table 2. In our case the CT data does not contain any significant structural information in the frequencies over *π*/2 in the spatial dimensions, the volumes are therefore lowpass filtered and then downsampled a factor 2 in *x*, *y*, *z*. When the local structure tensor has been estimated, it is lowpass filtered, with a separable lowpass filter of size 5 × 5 × 5 × 3, to improve the estimate in each time voxel and to make sure that the resulting reconstruction filter varies smoothly. Note that this smoothing does not decrease the resolution of the *image data*, but only the resolution of the *tensor field*. After the tensor mapping, the control tensor is interpolated to the original resolution of the CT data.

While the presented algorithm is straightforward to implement, spatial filtering with 11 reconstruction filters of size 11 × 11 × 11 × 11 (14 641 filter coefficients) applied to a dataset of the resolution 512 × 512 × 445 × 20 requires about 375 000 billion multiplications. This is the reason why the GPU is needed in order to do the 4D denoising in a reasonable amount of time.

### 2.9. Normalized Convolution

One of the main drawbacks of the presented algorithm is that, using standard convolution, the number of valid elements in the *z*-direction (i.e., slices) decreases rapidly. If the algorithm is applied to a dataset of the resolution 512 × 512 × 34 × 20, two slices are first lost due to the convolution with the lowpass filter of size 3 × 3 × 3. After the downsampling, there are 16 slices in the data. The monomial filters are of size 7 × 7 × 7 × 7, thereby only 10 of the filter response slices are valid. During the lowpass filtering of each structure tensor component, another four slices are lost and then another four are lost during lowpass filtering of the control tensor. The result is thus that only 2 valid slices are left after all the convolutions. The same problem could exist in the time dimension, but since the heart cycle is periodic it is natural to use circular convolution in the time direction, and thereby all the time points are valid.

The loss of valid slices can be avoided by using normalized convolution [34], both for the lowpass filtering of the data before downsampling and the lowpass filtering of the tensor components. In normalized convolution, a certainty is attached to each signal value. A certainty-weighted filter response cwr is calculated as

(25) $$\begin{array}{c} \text{cwr}=\frac{\left(c\cdot s\right)*f}{c*f}, \end{array}$$

where *c* is the certainty, *s* is the signal, *f* is the filter, · denotes pointwise multiplication, and ∗ denotes convolution. The certainty is set to 1 inside the data and 0 outside the data. Note that this simple version of normalized convolution (normalized averaging) can not be applied for the monomial filters and for the reconstruction filters, as these filters have both negative and positive coefficients. It is possible to apply the full normalized convolution approach for these filters, but it will significantly increase the computational load.

## 3. GPU Implementation

 In this section, the GPU implementation of the denoising algorithm will be described. The CUDA (compute unified device architecture) programming language by Nvidia [35], explained by Kirk and Hwu [36], has been used for the implementation. The Open Computing Language (OpenCL) [37] could be a better choice, as it makes it possible to run the same code on any hardware.

### 3.1. Creating 4D Indices

 The CUDA programming language can easily generate 2D indices for each thread, for example, by using Algorithm 1. To generate 3D indices is harder, as each thread block can be *three* dimensional but the grid can only be *two* dimensional. One approach to generate 3D indices is given in Algorithm 2. To generate 4D indices is even more difficult. To navigate in the 4D data, the 3D indexing approach described above is used, and the kernel is then called once for each time point.

### 3.2. Spatial versus FFT Based Filtering

Fast-Fourier-transform (FFT-) based filtering can be very efficient when large nonseparable filters of high dimension are to be applied to big datasets, but spatial filtering is generally faster if the filters are small or Cartesian separable. The main advantage with FFT-based filtering is that the processing time is the same regardless of the spatial size of the filter. A small bonus is that circular filtering is achieved for free. The main disadvantage with FFT-based filtering is however the memory requirements, as the filters need to be stored in the same resolution as the data, and also as a complex-valued number for each element.

To see which kind of filtering that fits the GPU best, both spatial and FFT-based filtering was therefore implemented. For filtering with the small separable lowpass filters (which are applied before the data is downsampled and to smooth the tensor components), only separable spatial filtering is implemented.

### 3.3. Spatial Filtering

Spatial filtering can be implemented in rather many ways, especially in four dimensions. One easy way to implement 2D and 3D filtering on the GPU is to take advantage of the cache of the texture memory and put the filter kernel in constant memory. The drawback with this approach is however that the implementation will be very limited by the memory bandwidth, and not by the computational performance. Another problem is that it is not possible to use 4D textures in the CUDA programming language. One would have to store the 4D data as one big 1D texture or as several 2D or 3D textures. A better approach is to take advantage of the shared memory, which increased a factor 3 in size between the Nvidia GTX 285 and the Nvidia GTX 580. The data is first read into the shared memory and then the filter responses are calculated in parallel. By using the shared memory, the threads can share the data in a very efficient way, which is beneficial as the filtering results for two neighbouring elements are calculated by mainly using the same data.

As multidimensional filters can be separable or nonseparable (the monomial filters and the reconstruction filters are nonseparable, while the different lowpass filters are separable) two different spatial filtering functions were implemented.

#### 3.3.1. Separable Filtering

Our separable 4D convolver is implemented by first doing the filtering for all the rows, then for all the columns, then for all the rods and finally for all the time points. The data is first loaded into the shared memory and then the valid filter responses are calculated in parallel. The filter kernels are stored in constant memory. For the four kernels, 16 KB of shared memory is used such that 3 thread blocks can run in parallel on each multiprocessor on the Nvidia GTX 580.

#### 3.3.2. Nonseparable Filtering

The shared memory approach works rather well for nonseparable 2D filtering but not as well for nonseparable 3D and 4D filtering. The size of the shared memory on the Nvidia GTX 580 is 48 KB for each multiprocessor, and it is thereby only possible to, for example, fit 11 × 11 × 11 × 9 float values into it. If the 4D filter is of size 9 × 9 × 9 × 9, only 3 × 3 × 3 × 1 = 27 valid filter responses can be generated for each multiprocessor. A better approach for nonseparable filtering in 4D is to instead use an optimized 2D filtering kernel, and then accumulate the filter responses by summing over the other dimensions by calling the 2D filtering function for each slice and each time point of the filter. The approach is described with the pseudocode given in Algorithm 3.

Our nonseparable 2D convolver first reads 64 × 64 pixels into the shared memory, then calculates the valid filter responses for all the 14 monomial filters or all the 11 reconstruction filters at the same time, and finally writes the results to global memory. Two versions of the convolver were implemented, one that maximally supports 7 × 7 filters and one that maximally supports 11 × 11 filters. The first calculates 58 × 58 valid filter responses, and the second calculates 54 × 54 valid filter responses. As 64 × 64 float values only require 16 KB of memory, three thread blocks can run at the same time on each multiprocessor. This results in 58 × 58 × 3 = 10092 and 54 × 54 × 3 = 8748 valid filter responses per multiprocessor. For optimal performance, the 2D filtering loop was completely unrolled by generating the code with a Matlab script.

The 14 monomial filters are of size 7 × 7 × 7 × 7, this would require 135 KB of memory to be stored as floats, but the constant memory is only 64 KB. For this reason, 7 × 7 filter coefficients are stored at a time and are then updated for each time point and for each slice. It would be possible to store 7 × 7 × 7 filter coefficients at a time, but by only storing 7 × 7 coefficients, the size of the filters (2.75 KB) is small enough to always be in the cache of the constant memory (8 KB). The same approach is used for the 11 reconstruction filters of size 11 × 11 × 11 × 11.

### 3.4. FFT-Based Filtering

 While the CUFFT library by Nvidia supports 1D, 2D, and 3D FFTs, there is no direct support for 4D FFTs. As the FFT is cartesian separable, it is however possible to do a 4D FFT by applying four consecutive 1D FFTs. The CUFFT library supports launching a batch of 1D FFTs, such that many 1D FFT's can run in parallel. The batch of 1D FFTs are applied along the first dimension in which the data is stored (e.g., along *x* if the data is stored as (*x*, *y*, *z*, *t*)). Between each 1D FFT, it is thereby necessary to change the order of the data (e.g., from (*x*, *y*, *z*, *t*) to (*y*, *z*, *t*, *x*)). The drawback with this approach is that the time it takes to change order of the data can be longer than to actually perform the 1D FFT. The most recent version of the CUFFT library supports launching a batch of 2D FFT's. By applying two consecutive 2D FFT's, it is sufficient to change the order of the data once, instead of three times.

A forward 4D FFT is first applied to the volumes. A filter is padded with zeros to the same resolution as the data and is then transformed to the frequency domain. To do the filtering, a complex-valued multiplication between the data and the filter is applied and then an inverse 4D FFT is applied to the filter response. After the inverse transform, a FFT shift is necessary; there is however no such functionality in the CUFFT library. When the tensor components and the denoised data are calculated, each of the four coordinates is shifted by using a help function, see Algorithm 4.

 As the monomial filters only have a real part or an imaginary part in the spatial domain, some additional time is saved by putting one monomial filter in the real part and another monomial filter in the imaginary part before the 4D FFT is applied to the zero-padded filter. When the complex multiplication is performed in the frequency domain, two filters are thus applied at the same time. After the inverse 4D FFT, the first filter response is extracted as the real part and second filter response is extracted as the imaginary part. The same trick is used for the 10 highpass reconstruction filters.

### 3.5. Memory Considerations

The main problem of implementing the 4D denoising algorithm on the GPU is the limited size of the global memory (3 GB in our case). This is made even more difficult by the fact that the GPU driver can use as much as 100–200 MB of the global memory. Storing all the CT data on the GPU at the same time is not possible, a single CT volume of the resolution 512 × 512 × 445 requires about 467 MB of memory if 32 bit floats are used. Storing the filter responses is even more problematic. To give an example, to store all the 11 reconstruction filter responses as floats for a dataset of the size 512 × 512 × 445 × 20 would require about 103 GB of memory. The denoising is therefore done for a number of slices (e.g., 16 or 32) at a time.

For the spatial filtering, the algorithm is started with data of the resolution 512 × 512 × 51 × 20 and is downsampled to 256 × 256 × 26 × 20. The control tensor is calculated for 256 × 256 × 20 × 20 time voxels, and the denoised data is calculated for 512 × 512 × 39 × 20 time voxels. To process all the 445 slices requires 12 runs.

For the FFT-based filtering, the algorithm is started with data of the resolution 512 × 512 × 31 × 20 and is downsampled to 256 × 256 × 16 × 20. The control tensor is then calculated for 256 × 256 × 10 × 20 time voxels, and the denoised data is calculated for 512 × 512 × 18 × 20 time voxels. To process all the 445 slices requires 26 runs.

To store the 10 components of the control tensor in the same resolution as the original CT data for one run with spatial filtering (512 × 512 × 39 × 20) would require about 12.2 GB of memory. As the control tensor needs to be interpolated a factor 2 in each spatial dimension, since it is estimated on downsampled data, another approach is used. Interpolating the tensor is a perfect task for the GPU, due to the hardware support for linear interpolation. The 10 tensor components, for one timepoint, are therefore stored in 10 textures and then the interpolation is done on the fly when the denoised data is calculated. By using this approach, only another 10 variables of the resolution 256 × 256 × 20 need to be stored at the same time.

Table 2 states the in and out resolution of the data, the used equations, and the memory consumption at each step of the denoising algorithm, for spatial filtering and FFT-based filtering. The out resolution refers to the resolution of the data that is valid after each processing step, as some data is regarded as non-valid after filtering operations. The reason why the memory consumption is larger for the FFT-based filtering is that the spatial filtering can be done for one slice or one volume at a time, while the FFT-based filtering has to be applied to a sufficiently large number of slices and time points at the same time. We were not able to use more than about 2 GB of memory for the FFT-based filtering; one reason for this might be that the CUFFT functions internally use temporary variables that use some of the memory. Since the source code for the CUFFT library is unavailable, it is hard to further investigate this hypothesis.

## 4. Data

The 4D CT dataset that was used for testing our GPU implementation was collected with a Siemens SOMATOM Definition Flash dual-energy CT scanner at the Center for medical Image Science and Visualization (CMIV). The dataset contains almost 9000 DICOM files and the resolution of the data is 512 × 512 × 445 × 20 time voxels. The spatial size of each voxel is 0.75 × 0.75 × 0.75 mm. During the image acquisition the tube current is modulated over the cardiac cycle with reduced radiation exposure during the systolic heart phase. Due to this, the amount of noise varies with time.

## 5. Results

### 5.1. Processing Times

A comparison between the processing times for our GPU implementation and for a CPU implementation was made. The used GPU was a Nvidia GTX 580, equipped with 512 processor cores and 3 GB of memory (the Nvidia GTX 580 is normally equipped with 1.5 GB of memory). The used CPU was an Intel Xeon 2.4 GHz with 4 processor cores and 12 MB of L3 cache, 12 GB of memory was used. All the implementations used 32 bit floats. The operating system used was Linux Fedora 14 64-bit.

For the CPU implementation, the OpenMP (open multiprocessing) library [38, 39] was used, such that all the 4 processor cores work in parallel. No other types of optimization for the CPU, such as SSE2, were used. We are fully aware of the fact that it is possible to make a much better CPU implementation. The purpose of this comparison is rather to give an *indication* of the performance of the CPU and the GPU. If the CPU code would be vectorized, the CPU processing times can be divided by a factor 3 or 4 (except for the FFT which already is very optimized).

The processing times are given in Tables 3, 4, 5, and 6. The CPU processing times for the spatial filtering are *estimates*, since it takes several days to run the algorithm on the whole dataset. The processing times for a multi-GPU implementation would scale rather linearly with the number of GPUs, since each GPU can work on different subsets of slices in parallel. As our computer contains three GPUs, all the processing times for the GPU can thereby be divided by a factor 3.

### 5.2. Denoising Results

**Figure 8:**
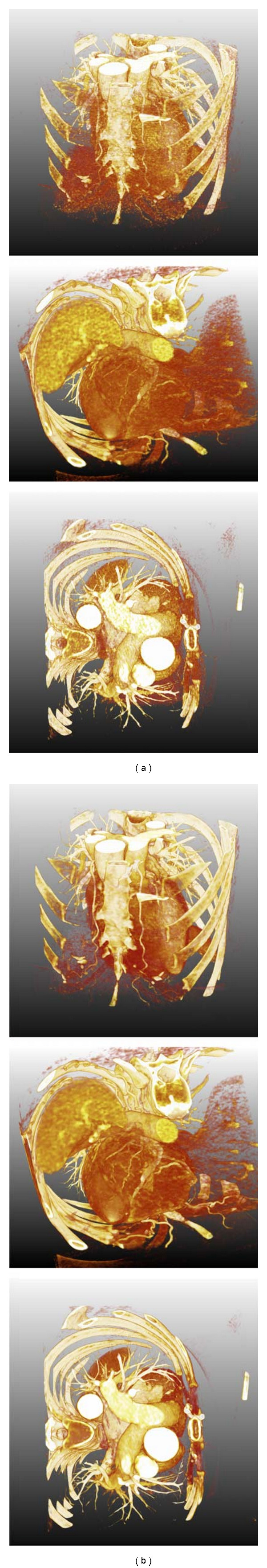
Three comparisons between original CT data (a) and denoised CT data (b). The parameters used for this denoising where *α* = 0.55, *β* = 1.5, and *σ* = 0.1 for the M-function.

To show the results of the 4D denoising, the original CT data was compared with the denoised data by applying volume rendering. The freely available MeVisLab software development program (http://www.mevislab.de/) was used. Two volume renderers, one for the original data and one for the denoised data, run at the same time and were synced in terms of view angle and transfer function.  Figure 8 shows volume renderings of the original and the denoised data for different time points and view angels. It is clear that a lot of noise is removed by the denoising, but since the denoising algorithm alters the histogram of the data, it is hard to make an objective comparison even if the same transfer function is applied.

A movie where the original and the denoised data is explored with the two volume renderers was also made. For this video, the data was downsampled a factor 2 in the spatial dimensions, in order to decrease the memory usage. The volume renderers automatically loop over all the timepoints. The video can be found at http://www.youtube.com/watch?v=wflbt2sV34M. 

 By looking at the video, it is easy to see that the amount of noise in the original data varies with time.

## 6. Discussion

 We have presented how to implement true 4D image denoising on the GPU. The result is that 4D image denoising becomes practically possible if the GPU is used and thereby the clinical value increases significantly.

### 6.1. Processing Times

To make a completely fair comparison between the CPU and the GPU is rather difficult. It has been debated [40] if the GPU speedups that have been reported in the literature are plausible or if they are the result of comparisons with unoptimized CPU implementations. In our opinion, the theoretical and practical processing performance that can be achieved for different hardware is not the only interesting topic. In a research environment, the *ratio* between the achievable processing performance and the time it takes to do the implementation is also important. From this perspective, we think that our CPU-GPU comparison is rather fair, since about the same time was spent on doing the CPU and the GPU implementation. The CUDA programming language was designed and developed for parallel calculations from the beginning, while different addons have been added to the C programming language to be able to do parallel calculations. While it is rather easy to make the CPU implementation multithreaded, for example, by using the OpenMP library, more advanced CPU optimization is often more difficult to include and often requires assembler programming.

While spatial filtering can be significantly slower than FFT-based filtering for nonseparable filters, there are some advantages (except for the lower memory usage). One is that a region of interest (ROI) can be selected for the denoising, compared to doing the denoising on the whole dataset. Another advantage is that filter networks [41, 42] can be applied, such that the filter responses from many small filters are combined to the same filter response as from one large filter. Filter networks can reduce the number of multiplications as much as a factor 5 in 2D, 25 in 3D and 300 in 4D [43]. To design and optimize a filter network however requires much more work than to optimize a single filter [33]. Another problem is that the memory usage increases significantly when filter networks are used, since many filter responses need to be stored in memory. Filter networks on the GPU is a promising area for future research.

From our results, it is clear that FFT-based filtering is faster than spatial filtering for large nonseparable filters. For data sizes that are not a power of two in each dimension, the FFT based approach might however not be as efficient. Since medical doctors normally do not look at 3D or 4D data as volume renderings, but rather as 2D slices, the spatial filtering approach however has the advantage that the denoising can be done for a region of interest (e.g., a specific slice or volume). It is a waste of time to enhance the parts of the data that are not used by the medical doctor. The spatial filtering approach can also handle larger datasets than the FFT-based approach, as it is sufficient to store the filter responses for one slice or one volume at a time. Recently, we acquired a CT data set with 100 time points, compared to 20 time points. It is not possible to use the FFT-based approach for this data set.

There are several reasons why the GPU speedup for the FFT-based filtering is much smaller than the GPU speedup for the spatial filtering. First, the CUFFT library does not include any direct support for 4D FFT's, and we had to implement our own 4D FFT as two 2D FFT's that are applied after each other. Between the 2D FFT's the storage order of the data is changed. It can take a longer time to change the order of the data than to actually perform the FFT. If Nvidia includes direct support for 4D FFT's in the CUFFT library, we are sure that their implementation would be much more efficient than ours. Second, the FFT for the CPU is extremely optimized, as it is used in a lot of applications, and our convolver for the CPU is not fully optimized. The CUDA programming language is only a few years old, and the GPU standard libraries are not as optimized as the CPU standard libraries. The hardware design of the GPUs also changes rapidly. Some work has been done in order to further optimize the CUFFT library. Nukada et al. [44, 45] have created their own GPU FFT library which has been proven to give better performance than the CUFFT library. They circumvent the problem of changing the order of the data and thereby achieve an implementation that is much more efficient. In 2008, their 3D FFT was 5-6 times faster than the 3D FFT in the CUFFT library. Third, due to the larger memory requirements of FFT-based filtering it is not possible to achieve an as big speedup for the GPU implementation as for the CPU implementation. If a GPU with a higher amount of global memory would have been used, the FFT-based implementation would have been more efficient.

### 6.2. 4D Image Processing with CUDA

As previously discussed in the paper, 4D image processing in CUDA is harder to implement than 2D and 3D image processing. There are, for example, no 4D textures, no 4D FFTs, and there is no direct support for 4D (or 3D) indices. However, since fMRI data also is 4D, we have previously gained some experience on how to do 4D image processing with CUDA [18–20]. The conclusions that we draw after implementing the 4D image denoising algorithm with the CUDA programming language is thus that CUDA is not perfectly suited for 4D image processing, but due to its flexibility, it was still possible to implement the algorithm rather easily.

### 6.3. True 5D Image Denoising

It might seem impossible to have medical image data with more than 4 dimensions, but some work has been done on how to collect 5D data [46]. The five dimensions are the three spatial dimensions and *two* time dimensions, one for the breathing rhythm and one for the heart rhythm. One major advantage with 5D data is that the patient can breathe normally during the data acquisition, while the patient has to hold its breath during collection of 4D data. With 5D data, it is possible to, for example, fixate the heart and only see the lungs moving, or fixate the lungs to only see the heart beating. If the presented algorithm would be extended to 5D, it would be necessary to use a total of 20 monomial filters and 16 reconstruction filters. For a 5D dataset of the size 512 × 512 × 445 × 20 × 20, the required number of multiplications for spatial filtering with the reconstruction filters would increase from 375 000 billion for 4D to about 119 million billion (1.19 · 10^17^) for 5D. The size of the reconstruction filter responses would increase from 103 GB for 4D to 2986 GB for 5D. This is still only *one* dataset for *one* patient, and we expect that both the spatial and the temporal resolution of all medical imaging modalities will increase even further in the future. Except for the 5 *outer* dimensions, it is also possible to collect data with more than one *inner* dimension. This is, for example, the case if the blood flow of the heart is to be studied. For flow data, a three-dimensional vector needs to be stored in each time voxel, instead of a single intensity value.

## 7. Conclusions

To conclude, by using the GPU, true 4D image denoising becomes practically feasible. Our implementation can of course be applied to other modalities as well, such as ultrasound and MRI, and not only to CT data. The short processing time also makes it practically possible to further improve the denoising algorithm and to tune the parameters that are used.

The elapsed time between the development of practically feasible 2D [2] and 3D [4] image denoising techniques was about 10 years, from 3D to 4D the elapsed time was about 20 years. Due to the rapid development of GPUs, it is hopefully *not* necessary to wait another 10–20 years for 5D image denoising.

## Figures and Tables

**Algorithm 1:**
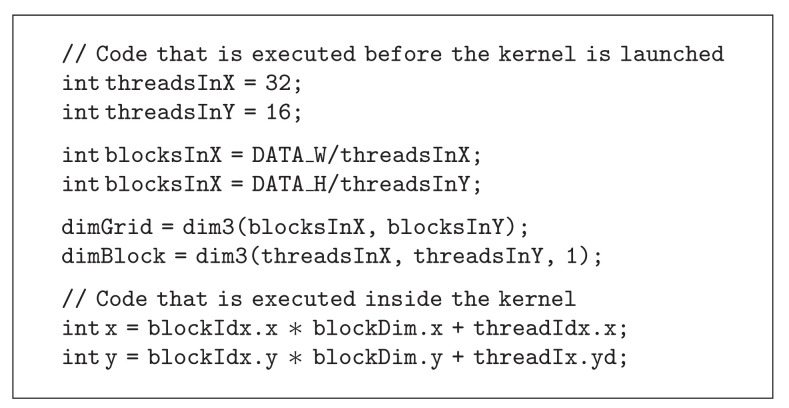


**Algorithm 2:**
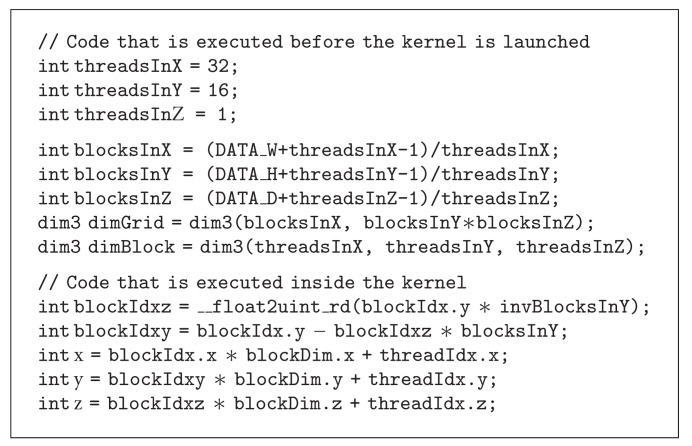


**Algorithm 3:**
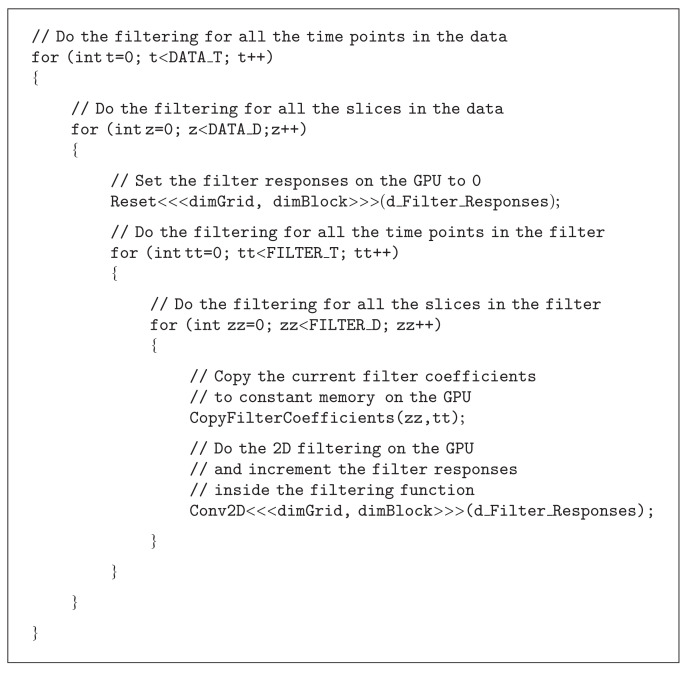


**Algorithm 4:**
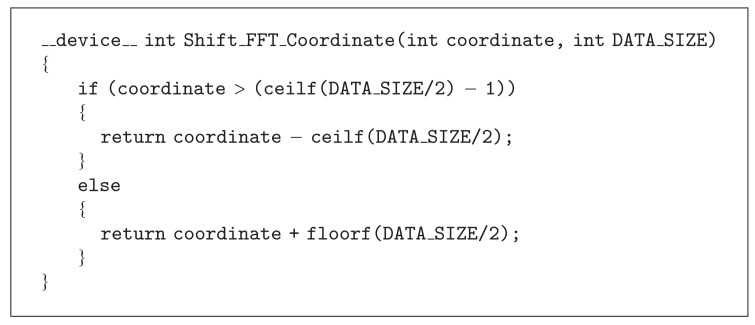


**Table 1:** Comparison between three Nvidia GPUs, from three different generations, in terms of processor cores, memory bandwidth, size of shared memory, cache memory, and number of registers; MP stands for multiprocessor and GB/s stands for gigabytes per second. For the GTX 580, the user can for each kernel choose to use 48 KB of shared memory and 16 KB of L1 cache or vice versa.

Property/GPU	9800 GT	GTX 285	GTX 580
Number of processor cores	112	240	512
Normal size of global memory	512 MB	1024 MB	1536 MB
Global memory bandwidth	57.6 GB/s	159.0 GB/s	192.4 GB/s
Constant memory	64 KB	64 KB	64 KB
Shared memory per MP	16 KB	16 KB	48/16 KB
Float registers per MP	8192	16384	32768
L1 cache per MP	None	None	16/48 KB
L2 cache	None	None	768 KB

**Table 2:** The table shows the in and out data resolution, the used equations and the memory consumption for all the processing steps for spatial filtering (SF) and FFT-based filtering (FFTBF). Note that the driver for the GPU is stored in the global memory, and it normally requires 100–200 MB.

Processing step	Resolution, SF	Memory consumption, SF	Resolution, FFTBF	Memory consumption, FFTBF
Lowpass filtering and downsampling of CT volumes	in 512 × 512 × 51 × 20	406 MB	in 512 × 512 × 31 × 20	294 MB
	out 256 × 256 × 26 × 20		out 256 × 256 × 16 × 20	

Filtering with 14 monomial filters and calculating the local structure tensor ((10), (11))	in 256 × 256 × 26 × 20	1376 MB	in 256 × 256 × 16 × 20	1791 MB
	out 256 × 256 × 20 × 20		out 256 × 256 × 10 × 20	

Lowpass filtering of the local structure tensor components (normalized convolution, (25))	in 256 × 256 × 20 × 20	1276 MB	in 256 × 256 × 10 × 20	720 MB
	out 256 × 256 × 20 × 20		out 256 × 256 × 10 × 20	

Calculating the tensor magnitude and mapping it with the M-function ((17), (18), (19), (13))	in 256 × 256 × 20 × 20	1376 MB	in 256 × 256 × 10 × 20	770 MB
	out 256 × 256 × 20 × 20		out 256 × 256 × 10 × 20	

Mapping the local structure tensor to the control tensor ((20), (22), (23))	in 256 × 256 × 20 × 20	1376 MB	in 256 × 256 × 10 × 20	770 MB
	out 256 × 256 × 20 × 20		out 256 × 256 × 10 × 20	

Lowpass filtering of the control tensor components (normalized convolution, (25))	in 256 × 256 × 20 × 20	1476 MB	in 256 × 256 × 10 × 20	820 MB
	out 256 × 256 × 20 × 20		out 256 × 256 × 10 × 20	

Filtering with 11 reconstruction filters, interpolating the control tensor on the fly, and calculating the denoised data (24)	in 512 × 512 × 51 × 20	2771 MB	in 512 × 512 × 16 × 20	2110 MB
	out 512 × 512 × 39 × 20		out 512 × 512 × 6 × 20 (three rounds×6 slices = 18 denoised slices in total)	

**Table 3:** Processing times for filtering with the 14 monomial filters of size 7 × 7 × 7 × 7 and calculating the 4D tensor for the different implementations. The processing times for the GPU do not include the time it takes to transfer the data to and from the GPU.

Data size	Spatial filtering CPU	Spatial filtering GPU	GPU speedup	FFT filtering CPU	FFT filtering GPU	GPU speedup
128 × 128 × 111 × 20	17.3 min	5.7 s	182	25 s	1.8 s	13.9
256 × 256 × 223 × 20	2.3 h	36.0 s	230	3.3 min	14.3 s	13.9

**Table 4:** Processing times for lowpass filtering the 10 tensor components, calculating *γ* and mapping the structure tensor to the control tensor for the different implementations. The processing times for the GPU do not include the time it takes to transfer the data to and from the GPU.

Data size	CPU	GPU	GPU speedup
256 × 256 × 223 × 20	42 s	1.0 s	42
512 × 512 × 445 × 20	292 s	7.3 s	40

**Table 5:** Processing times for filtering with the 11 reconstruction filters of size 11×11×11×11 and calculating the denoised data for the different implementations. The processing times for the GPU do NOT include the time it takes to transfer the data to and from the GPU.

Data size	Spatial filtering CPU	Spatial filtering GPU	GPU speedup	FFT filtering CPU	FFT filtering GPU	GPU speedup
256 × 256 × 223 × 20	7.5 h	3.3 m	136	5.6 min	1.1 min	5.1
512 × 512 × 445 × 20	2.5 days	23.9 m	150	45 min	8.6 min	5.2

**Table 6:** Total processing times for the complete 4D image denoising algorithm for the different implementations. The processing times for the GPU DO include the time it takes to transfer the data to and from the GPU.

Data size	Spatial filtering CPU	Spatial filtering GPU	GPU speedup	FFT filtering CPU	FFT filtering GPU	GPU speedup
256 × 256 × 223 × 20	7.8 h	3.5 m	133	6.7 m	1.2 m	5.6
512 × 512 × 445 × 20	2.6 days	26.3 m	144	52.8 m	8.9 m	5.9
